# Veterinary College Notes

**Published:** 1890-02

**Authors:** 


### VETERINARY COLLEGE NOTES.

*Ontario Veterinary College.*—The Agriculture and Arts Association of Ontario, at its late meeting in Toronto, passed the following resolution, which gives some idea of what is thought of the labors and efforts of Prof. Smith in connection with veterinary matters :—

"That the Council of this Association, having visited and inspected the handsome new Veterinary College created by Dr. Smith, desires to record its pleasure and satisfaction at the wonderful progress made by this institution."

The motion further went on to say that it was "a matter of special gratification to the Council to know that an institution which was inaugurated under its auspices, and whose graduates still receive their diplomas from this Council, has been attended with such success."

The examiners appointed by the Board of the Agriculture and Arts Association concluded the Christmas examinations of the College on Friday, December 20th. The following gentlemen were awarded diplomas :

Walter Amos, Frank H. Anderson, Joseph F. Black, Henry Bradshaw, David J. Carson, Montague A. Davies, Frederick H. P. Edwards, Robert Fawns, John Fyfe, Ulysses S. G. Faling, Willard H. Harris, Charles H. Hunt, Thomas H. Hassard, James Hanson, Charles A. Howson, William D. Hammond, James J. Joy, James H. Johnson, Robert Johnston, James J. Karr, William Keogh, Patrick J. Lynchke, Edmund Latousell, Arnold F. Lees, John Martin, W. Sanford Niles, Jefferson Pulford, James C. Sharp, Moses Sinclair, Elias P. Smith, Robert M. Thompson, T. James Todd, Michael Whalen, James N. Wilkinson.

The following were granted primaries :—

In anatomy, William S. Lyons, William Routledge, Edwin C. Radley ; in materia medica, William J. Gerrow, Charles E. Hatch, J. Gordon McPherson, Andrew L. Milroy, John H. C. Todd.

The examinations were conducted in the usual strict and impartial manner by the Board of Examiners appointed by the Council of Agriculture and Arts, who have acted in this capacity for some years back. The success of this institution must be attributed to the thorough nature of the teachings imparted by the principal, Prof. Smith, and his assistant teachers. This is demonstrated by the successful practice of its graduates, who are scattered over all parts of the North American continent.

With the advent of the new year the new college just erected will be completed and occupied. This building is furnished with every convenience, lecture rooms, class rooms, etc., for the teaching of all branches of veterinary science, and it is expected that notwithstanding the extensive requirements for accommodation, the building will contain within itself halls and class rooms sufficient in capacity to meet every demand.

*The Baltimore University School of Veterinary Science*, which opened October 1st, 1889, shows among the veterinarians who are members of the faculty quite a number of government officials, who should make sanitary police a strong element of their instruction.

The list includes Prof. H. W. Wray, D.V.S., State Veterinarian; Veterinary Inspector, B. A. I.; Prof. Faville, D.V.M., B. Sc., Chief Inspector in the B. A. I., Maryland Division; Prof. Ward, Ex-State V. S., M.R.C.V.S., 1865, F.R.C.V.S., 1878, by Examination Cattle Pathologist "L. S. Journal," "Artemus Secundus."

---

*New York College of Veterinary Surgeons.*—The following new members have been added to the faculty: Prof. H. M. Biggs (Physiology); Prof. H. D. Gill (Theory and Practice); Prof. W. R. Ballou (Anatomy); Prof. Byron (Bacteriology); Prof. F. F. Reyling (Practical Histology and Microscopy); Prof. R. J. Carlisle (Pathology). They fill vacancies caused by the resignations of: Prof. Knipe, (Anatomy); Dr. R. A. McLean (Theory and Practice); Prof. L. McLean (Bovine Pathology); Dr. P. C. Hoag (Physiology); Dr. J. A. Breakell (Assistant Bovine Pathology).

The lecture room has been refitted and additions have been made in the operating room, chemical appliances and room for practical microscopy.

### NECROLOGY.

